# Clinical Value of Nomogram Model based on Multimodality Ultrasound Image Characteristics Differentiating Benign and Malignant Breast Masses

**DOI:** 10.2174/0115734056378619250722034152

**Published:** 2025-08-04

**Authors:** Jiaxin Yan, Jianting Zheng, Shurong Chen, Jiahua Zhao, Yangfan Han, Bo Liang

**Affiliations:** 1 Department of Medical Ultrasound, Affiliated Hospital 2 of Nantong University, Nantong First People's Hospital, Nantong, Jiangsu Province, China

**Keywords:** Nomogram model, Multimodality ultrasound image, Malignant breast masses, Benign breast masses, Elasticity scoring

## Abstract

**Introduction:**

Finding a convenient, accurate, and non-invasive method to differentiate between benign and malignant breast masses is especially important for clinical practice, and this study aimed to explore the clinical value of Nomogram model based on multimodality ultrasound image characteristics and clinical baseline data for detecting benign and malignant breast masses.

**Methods:**

A retrospective analysis of the clinical data and ultrasound imaging characteristics of 132 patients with breast masses. Data were randomly divided into a training set (92 cases) and a validation set (40 cases) in a ratio of 7:3. Logistic regression was applied to the training set data to analyze risk factors related to malignant breast masses and to construct a Nomogram model. Clinical applicability of the model was evaluated and validated.

**Results:**

In training set, ROC cure analysis results showed that AUC of Nomogram model constructed with CA15-3, CA125, E_max_, E_sd_, Ratio of Elastic Moduli, Elasticity Scoring, blurry boundaries, irregular shape, penetrating vessels, and stiff rim sign was 1.00 (95%CI: 0.99-1.00), Hosmer-Lemeshow goodness-of-fit test result showed predicted curve closely aligns with ideal curve, and DCA showed that Nomogram model exhibited high net benefits across multiple thresholds. The clinical applicability of the Nomogram model was also confirmed with consistent results in the validation set.

**Discussion:**

In this study, we constructed a Nomogram model using risk factors associated with malignant breast masses, and the model showed good clinical applicability in distinguishing benign and malignant breast masses. However, this study is a single-center study, and the sample size of the dataset is relatively small, which, to some extent, limits the breadth and depth of validation.

**Conclusion:**

The Nomogram model built on multimodal ultrasound imaging features and clinical data demonstrates a strong discriminative ability for malignant breast masses, allowing patients to achieve a significant net benefit.

## 1 INTRODUCTION

Breast masses are a common type of breast lesion, and their occurrence is gradually becoming more prevalent among younger individuals [1]. In particular, malignant breast masses have shown a yearly increase in incidence in recent years, causing significant physical and psychological harm to women, as well as impacting their quality of life [2]. Based on their nature, breast masses can generally be classified into benign tumors and malignant tumors. Fibroadenomas are the most common benign tumors, while breast cancer is the most common malignant tumor [3]. Breast cancer is one of the most common cancers worldwide, with varying incidence rates across different regions and populations [4]. Generally, the incidence of breast cancer in women varies due to factors such as age, genetic predisposition, lifestyle, and environmental influences [5]. The International Agency for Research on Cancer conducted a statistical analysis of data related to common tumors and found that in 2020, the number of new cases of breast cancer in women surpassed that of lung cancer, making it the most common cancer globally [6]. Breast cancer became the cancer with the highest incidence rate among women in both the United States and China. In China, new cases of breast cancer accounted for approximately 19.6% of all cancers diagnosed in women, yet the five-year relative survival rate is relatively high, reaching 82% [7].

To improve the detection rate of malignant breast tumors, imaging-related examinations are particularly important. In recent years, imaging technology has developed rapidly, with commonly used clinical imaging techniques including mammography, magnetic resonance imaging (MRI), and ultrasound examination. Ultrasound examination is a routine procedure in breast assessments and primarily includes techniques such as 2D ultrasound, color Doppler, and elastography. The definitive gold standard for diagnosing breast tumors is pathological diagnosis. Ultrasound examination is a non-invasive method that does not cause additional discomfort to patients. It allows for real-time observation of the shape, borders, and blood flow of masses, which helps in determining their nature [8-10]. Compared to other imaging techniques (such as MRI), ultrasound exams are less expensive. Additionally, they do not involve the use of radioactive materials, making them suitable for frequent assessments. However, ultrasound can only provide imaging information and cannot directly evaluate the histological characteristics of the masses. When imaging studies cannot determine the benign or malignant nature of a breast tumor, clinical practice often requires ultrasound-guided fine-needle biopsy or surgical procedures to obtain tissue for pathological examination. However, these invasive procedures can cause varying degrees of harm to patients. Additionally, unsatisfactory tissue samples can still lead to false-negative pathological results [11]. Therefore, finding a convenient, accurate, and non-invasive method to differentiate between benign and malignant breast tumors is especially important for clinical practice. Elastography (such as strain elastography and shear wave elastography) provides mechanical property information of lesions by quantifying tissue stiffness. Malignant lesions usually show higher elasticity modulus values (such as E_max_, E_mean_) due to stromal fibrosis and cell proliferation [12]. Meanwhile, serum biomarkers (such as SYPL1, CA15-3, *etc.*) can reflect tumor-related molecular biological changes, such as cell proliferation, angiogenesis, or immune evasion [13]. The combination of the two can capture the structural heterogeneity and molecular characteristics of lesions simultaneously, reducing the risk of false negatives or false positives associated with single-modality approaches. A study has shown that the combination of elastography and serum molecular markers detection can increase the AUC value for breast cancer diagnosis [14]. This study aimed to explore the clinical value of the Nomogram model based on multimodality ultrasound image characteristics and clinical baseline data, detecting benign and malignant breast masses.

## 2 METHODS

### 2.1 Patients

A retrospective analysis of the clinical data and ultrasound imaging characteristics of patients with diagnosed breast masses at our hospital from January 2021 to June 2024. Inclusion criteria: (1) Female and Age ≥ 18 years; (2) Complete ultrasound examination data; (3) Unifocal mass-type lesions: The term refers to the presence of only one solitary, localized mass-like lesion within the breast, with no other lesions on the same side or the opposite side. On imaging, it appears as a distinct “mass” (*i.e*., a three-dimensional space-occupying lesion), rather than a non-mass-like lesion (such as architectural distortion or scattered microcalcifications; (4) Clear pathological diagnosis of fibroadenomas or breast cancer; (5) Complete clinical data. Exclusion criteria: (1) Breast puncture biopsy or surgery before ultrasound examination; (2) History of other malignant tumors; (3) Poor quality of ultrasound images; (4) Incomplete case data; (5) History of breast prosthesis implantation; (6) Other breast diseases. A total of 132 patients were enrolled based on the inclusion and exclusion criteria. They were randomly divided into a training set (92 cases) and a validation set (40 cases) in a 7:3 ratio with the random number table method. The flowchart of patient inclusion/exclusion is shown in Fig. (**1**). Detailed case information was provided in Table **1**. All patients signed informed consent forms.

### 2.2 Ultrasound Examination Method

The examinations were conducted using the Resona I9T color Doppler ultrasound diagnostic device from Shenzhen Mindray Bio-Medical Electronics Co., Ltd., equipped with a 3-14 MHz linear array transducer and STE imaging software. Three experienced ultrasound physicians performed the examinations (Fleiss’ κ=0.68, ICC=0.72).

During the routine ultrasound examination, patients were positioned supine to fully expose the breast, a coupling agent was applied, and the transducer was placed in complete contact with the skin to visualize the mass at the center of the screen. The machine's preset “breast” mode was selected, and the maximum vertical diameter of the mass was measured on the longitudinal gray-scale image. In the perpendicular transverse plane, the horizontal and anteroposterior diameters were measured. The measurements were recorded as maximum vertical diameter * horizontal diameter * anteroposterior diameter (mm), along with observations of the lesion's morphology, blood flow, and margins.

Shear Wave Elastography Examination: The mode was switched to elastography imaging. No additional pressure was applied, and the transducer was fixed while adjusting the sampling box size to optimally cover the mass and its surrounding area. Patients were instructed to hold their breath to maintain a clear and stable image. Images were stored when the reliability index of the STE image was greater than 95%. The region of interest (ROI) for the mass was manually delineated, and the quantitative elasticity imaging of the surrounding glandular tissue (0.5-3 mm) was automatically measured. The elasticity parameters for the mass included the maximum Young’s modulus hardness value (E_max_), mean value (E_mean_), minimum value (E_min_), and standard deviation (E_sd_). For the surrounding glandular tissue, the elasticity parameters included the maximum Young’s modulus hardness value of the 2 mm shell (S_max_), mean value (S_mean_), minimum value (S_min_), and standard deviation (S_sd_). The average values were taken to reduce measurement errors.

### 2.3 Observation Indicators

Clinical data included age, body mass index (BMI), serum tumor markers, *etc.*

Ultrasound imaging features: maximum diameter, tumor depth, heterogeneous echogenicity, blurry boundaries, irregular shape, calcification, liquefactive necrosis, penetrating vessel, peak systolic velocity, resistance index, elasticity scoring (was assessed with Tissue Strain Ratio) [15], and stiff rim sign (SRS).

### 2.4 Statistical Processing

Measurement data that conform to a normal distribution as determined by the Kolmogorov-Smirnov D test are expressed as (mean±SD). Independent t-tests are used for intergroup comparisons. Data that do not conform to a normal distribution are expressed as median (interquartile range), with Mann-Whitney U rank-sum tests used for intergroup comparisons. Count data are expressed as composition ratios, with chi-square tests for intergroup comparisons. A power analysis by PASS 11.0 showed that the power with 132 cases in two groups for observation indicators achieves 100% at a significance level of 5%. The data were randomly divided into training and validation sets in a 7:3 ratio. Indicators showing differences between benign and malignant masses in the training set were entered into a binary logistic regression equation for unconditional univariate analysis to identify associated risk factors for malignant masses. The identified risk factors were then included in a binary logistic regression equation for unconditional multivariate analysis to determine independent high-risk factors for malignant masses. A nomogram prediction model was constructed using the relevant risk factors in the binary logistic regression equation, evaluating the clinical validity of the model in predicting malignant masses using methods such as Receiver Operating Characteristic (ROC) curves, Hosmer-Lemeshow goodness-of-fit test, calibration curves, and decision curve analysis (DCA). Finally, the clinical applicability of the model was validated in the validation set. A p-value of less than 0.05 was considered statistically significant. Data processing and analysis were performed using R version 4.4.0, along with Zstats 1.0 (www.zstats.net).

## 3 RESULTS

### 3.1 Balance Test of the Training Set and Validation Set

The analysis results showed that there were no significant differences in the clinical baseline data and imaging data between the training set and the validation set (Table **1**).

### 3.2 Comparison of Clinical Baseline Data and B Ultrasound Imaging Characteristics of Benign and Malignant Breast Masses in the Training Set

The analysis results of the training set showed that in patients with malignant breast masses, the serum levels of CA15-3 and CA125 were significantly higher than those in the benign group. In terms of ultrasound imaging, the E_max_, E_sd_, Ratio of Elastic Moduli, and Elasticity Scoring of malignant breast masses were significantly higher than in the benign group. Malignant breast masses often exhibited characteristics such as blurry boundaries, irregular shape, penetrating vessels, and SRS (Table **2**).

### 3.3 Risk Factors Associated with Malignant Breast Masses in the Training Set

The clinical and imaging indicators that showed differences between the benign and malignant groups were substituted into the Logistic regression equation. The results indicated that elevated serum levels of CA15-3 and CA125, higher values of E_max_, E_sd_, Ratio of Elastic Moduli, and Elasticity Scoring, as well as features such as blurry boundaries, irregular shape, penetrating vessels, and SRS are associated risk factors for malignant breast masses (Table **3**). When all the above indicators were included in the Logistic regression equation, the results showed that a higher value of Elasticity Scoring is an independent risk factor for malignant breast masses (Table **4**).

### 3.4 Construction and Evaluation of the Nomogram Model for the Training Set

**Fig. (2):**
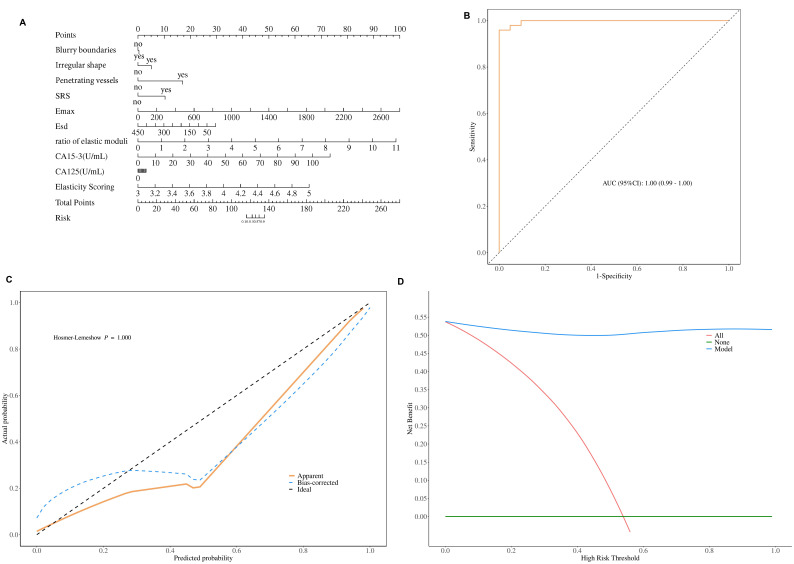
Evaluation of the clinical value of the Nomogram prediction model in the training set. (**A**) Visualization of the constructed prediction model featuring independent risk factors using a Nomogram. (**B**) Results from the ROC analysis. (**C**) Outcomes from the calibration curve. (**D**) Findings from the DCA.

According to the logistic regression analysis results, the Nomogram prediction model was constructed with risk factors. The equation is as follows: Logit (P) = 2.24×blurry boundaries+ 0.93×irregular shape + 2.91×penetrating vessels+1.69×SRS+0.01×E_max_+0.01×E_sd_+1.20×Ratio of Elastic Moduli+0.12×CA15-3+0.07×CA125+4.99×Elasticity Scoring. The constructed prediction model was visualized using a Nomogram (Fig. **2A**).

The ROC curve analysis results showed that Area Under the Curve (AUC) of Nomogram model was 1.00 (95%CI: 0.99-1.00), and accuracy, sensitivity, and specificity was 0.98, 1.00, 0.96 respectively (Table **5**, Fig. **2B**). The Nomogram prediction model demonstrated good discrimination. The Hosmer-Lemeshow goodness-of-fit test result showed that χ2=0.615, P=1.000, the predicted curve closely aligns with the ideal curve (Fig. **2C**), indicating that the model has high accuracy in its predictions and effectively reflects the probability of actual events occurring. The DCA showed that the Nomogram model exhibited high net benefits across multiple thresholds for malignant breast masses (Fig. **2D**), indicating that the Nomogram model has good potential for application in clinical decision-making.

### 3.5 Validation of the Nomogram Model 's Clinical Applicability in the Validation Set

**Fig. (3):**
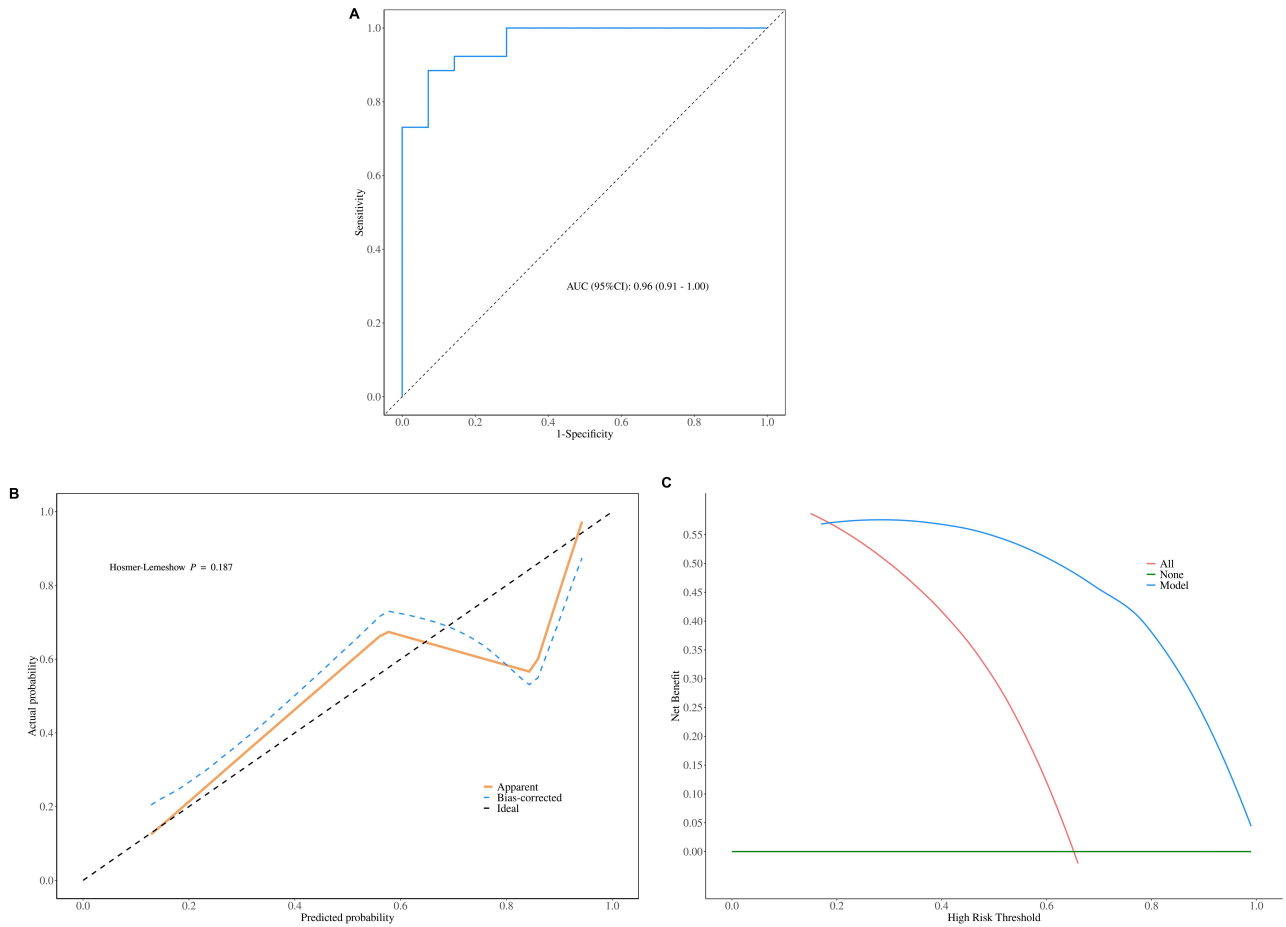
3 Verification of the clinical effectiveness of the nomogram predictive model in the validation set. (**A**) ROC results. (**B**) Calibration curve results. (**C**) DCA results.

The ROC cure analysis results showed that AUC of Nomogram model was 0.96 (95%CI: 0.91-1.00), and accuracy, sensitivity, specificity was 0.88, 0.86, 0.88, respectively (Table **5**, Fig .**3A**). The Hosmer-Lemeshow goodness-of-fit test result showed that χ2=11.272, P=0.187, predicted curve closely aligns with the ideal curve (Fig. **3B**). The DCA showed that the Nomogram model exhibited high net benefits from 0.2-0.99 thresholds for malignant breast masses (Fig. **3C**). The clinical applicability of Nomogram model was also confirmed with consistent results in the validation set.

## 4 DISCUSSION

Breast cancer is a significant global public health issue that poses a serious threat to human health and contributes to a substantial disease burden [16]. In 2022, there were 2.3089 million new cases of breast cancer reported worldwide, making it the second most prevalent malignant tumor; there were 665,700 deaths due to breast cancer, ranking fourth among all malignancies [7]. In China, 357,200 new cases of breast cancer were reported in 2022, placing it sixth among all malignant tumors, with 75,000 deaths, ranking seventh [17]. The five-year survival rate for early-stage breast cancer exceeds 95%, whereas the survival rate for late-stage metastatic breast cancer is below 20% [18]. Therefore, early diagnosis of breast cancer is crucial for improving patient survival rates and enhancing prognosis.

The examination of breast cancer includes laboratory tests, imaging studies, and pathological assessments. Common tumor markers used in laboratory tests include CA15-3, carcinoembryonic antigen (CEA), and CA125 [19]; however, their sensitivity in diagnosing breast cancer is relatively low, and these tumor markers can also be elevated in other tumor lesions. The results of this study indicate that CA 15-3 and CA 125 are associated risk factors for malignant breast masses. Conventional ultrasound examination is a common non-invasive method for breast assessment, allowing real-time observation of the morphology, boundaries, and blood flow of lesions, which helps in determining their nature. In this study, we found malignant breast masses often exhibited characteristics such as blurry boundaries, irregular shape, and penetrating vessels, and logistic regression analysis results showed they were associated with risk factors for malignant breast masses. This is consistent with the findings of other researchers [9, 20, 21]. The unclear boundaries and irregular shape of malignant breast masses are due to the infiltration and destruction of surrounding tissues by tumor cells. These characteristics hold significant diagnostic value in breast ultrasound examinations, indicating the potential malignancy of the lesion [22]. However, it is important to note that imaging studies alone cannot definitively diagnose the nature of a breast mass; a final diagnosis requires integration of other clinical information and pathological assessments. As a type of neovascularization, penetrating vessels provide essential nutrients and oxygen to tumors, facilitating their growth and spread. In breast ultrasound examinations, the presence of feeding vessels is an important diagnostic clue for breast cancer [23, 24]. Compared to traditional two-dimensional ultrasound, ultrasound shear wave elastography can more vividly display the elasticity information of breast masses. It utilizes the differences in elastic coefficients between different tissues, with varying degrees of deformation under external pressure, transforming the changes in echo signal movement before and after compression into real-time color images. These images can be quantitatively assessed using software, providing an accurate and objective evaluation that intuitively reflects the hardness distribution of tissues, enabling doctors to make more precise judgments about the nature of the lesions [25-27]. In this study, the E_max_, E_sd_, Ratio of Elastic Moduli, and Elasticity Scoring of malignant breast masses were significantly higher than in the benign group. Malignant breast masses often exhibited SRS. Logistic regression analysis results showed they were associated risk factors for malignant breast masses, and a higher value of Elasticity Scoring is an independent risk factor for malignant breast masses.

A nomogram is a commonly used clinical prediction model that visually integrates the scores of various predictive indicators and presents them in a coordinate system. The nomogram prediction model is based on multiple predictive factors, which can include clinical baseline indicators, pathological characteristics, and relevant laboratory or imaging examination indicators. These factors are assigned different scores to reflect their importance in the assessment. Nomogram models can assist clinicians and researchers in quickly and intuitively evaluating the relevant risks for patients [28-30]. Nomograms are considered a part of Explainable Artificial Intelligence (XAI). Nomograms, as an “explainable by-design” model, can intuitively display the contribution of multimodal features to the prediction results, meeting the mandatory requirement for model explainability in clinical scenarios [31, 32]. XAI methods can enhance model credibility and diagnostic performance. Recent studies have shown that XAI not only verifies the rationality of models but also improves diagnostic accuracy by 5-8% through a feedback mechanism for feature correction [33]. Current research emphasizes that XAI is a necessary technical approach to meet the “right to explanation” requirements of medical data regulations such as GDPR [34]. Particularly in the field of breast cancer diagnosis, explainable decision-making basis can effectively avoid algorithmic liability disputes in medical disputes [35]. According to the latest clinical research, 85% of radiologists believe that XAI visualization can enhance trust in AI systems. Multicenter trials have shown that such methods can increase physician adoption rates from 62% to 89% [36]. In this study, we constructed a Nomogram model using the abovementioned risk factors associated with malignant breast masses in the training set. According to the nomogram, E_max_, E_sd_, the ratio of elastic moduli, CA15-3, and Elasticity Scoring have relatively high predictive score contributions. The ROC cure analysis results demonstrated Nomogram prediction model had good discrimination (AUC 1.00, accuracy 0.98, sensitivity 1.00, specificity 0.96), AUC=1.00, there may be overfitting in the training set. This suggests that the model may be overfitting to the noise in the training data. Further validation is needed in subsequent studies. The Hosmer-Lemeshow goodness-of-fit test result showed predicted curve closely aligns with the ideal curve, indicating that the model has high accuracy in its predictions and effectively reflects the probability of actual events occurring. The DCA showed patients can obtain net benefits when the model's threshold is between 0.00 and 100. The clinical applicability of the Nomogram model was also confirmed with consistent results in the validation set. Yan et al, established a Nomogram based on imaging aspects of conventional ultrasonography and contrast-enhanced ultrasound to identify benign from malignant breast lesions. They found the nomogram model demonstrated satisfactory discriminative function; in addition, the nomogram model showed good consistency and clinical potential according to the calibration curve and DCA [37]. Our model's performance is essentially consistent with theirs. However, this study is a single-center study, and the sample size of the dataset is relatively small, which, to some extent, limits the breadth and depth of validation. Therefore, in future research, we plan to further increase the sample size to more comprehensively verify the accuracy and reliability of the model.

## CONCLUSION

In a summary, the Nomogram model built on multimodal ultrasound imaging features and clinical data demonstrates a strong discriminative ability for malignant breast masses, allowing patients to achieve a significant net benefit. The nomogram model has good potential for application in clinical decision-making. However, this study is a single-center, small-sample research, and the clinical applicability of this prediction model has not been externally validated. This needs to be confirmed in our future research.

## AUTHORS’ CONTRIBUTIONS

The authors confirm their contribution to the paper as follows: B.L.: Study conception and design: J.Z.: Methodology: J.Z.: Data collection: Y.H.: Data Curation: S.C.: Analysis and interpretation of results: J.Y.: Draft manuscript. All authors reviewed the results and approved the final version of the manuscript.

## Figures and Tables

**Fig. (1):**
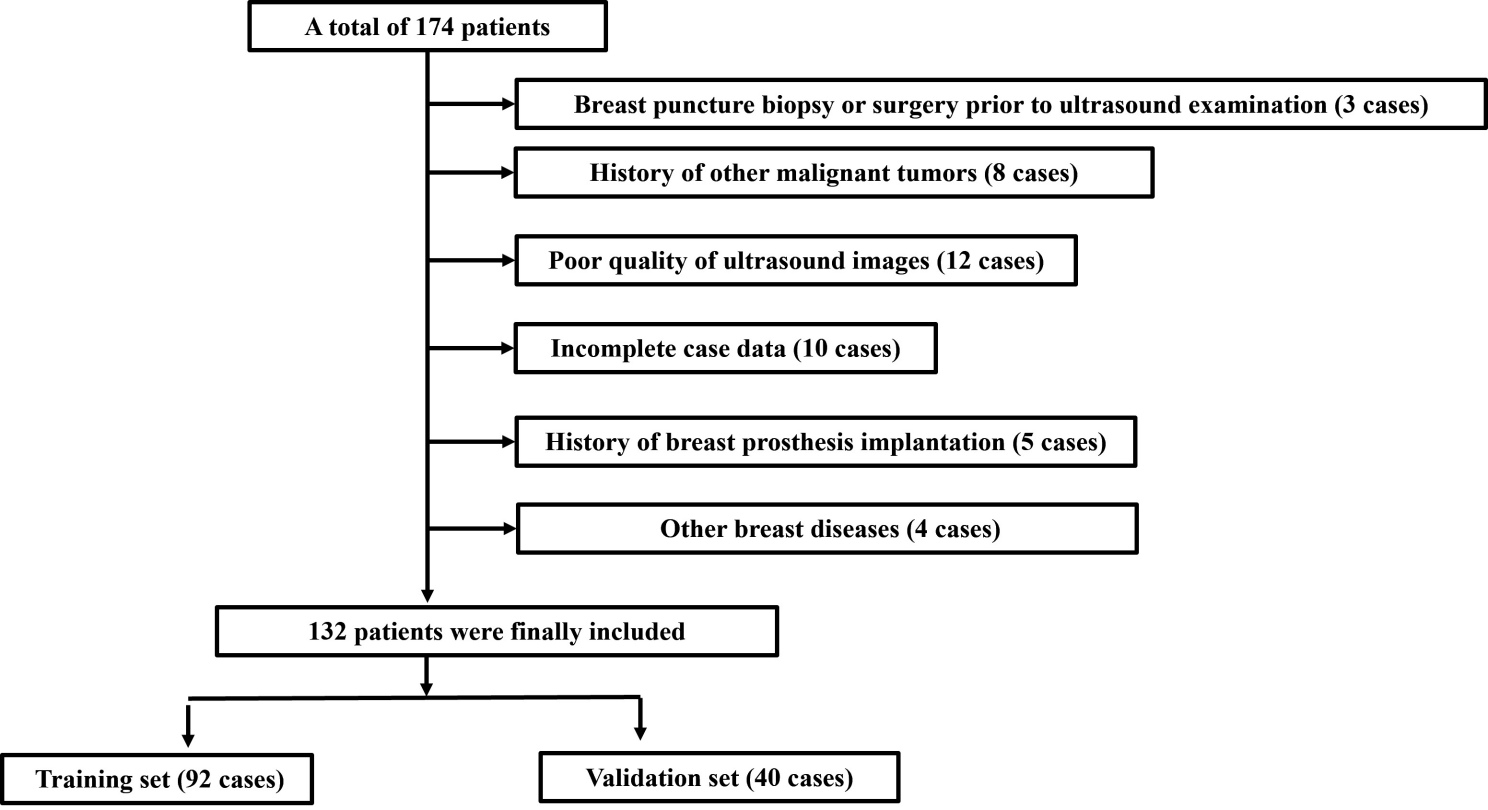
The flowchart of patient inclusion/exclusion.

**Table 1:** Balance test of the training set and validation set.

**Variables**	**Total (n = 132)**	**Test (n = 40)**	**Train (n = 92)**	**Statistic**	**P**
Age, mean ± SD	49.68 ± 11.74	48.20 ± 11.71	50.33 ± 11.76	t=-0.96	0.341
BMI, mean ± SD	22.81 ± 2.34	22.62 ± 2.40	22.90 ± 2.32	t=-0.64	0.526
CA199(U/mL), M (Q_1_, Q_3_)	7.85 (5.52, 11.53)	7.42 (5.71, 10.27)	7.90 (5.30, 12.05)	Z=-0.49	0.626
AFP(ng/mL), M (Q_1_, Q_3_)	2.59 (2.12, 3.43)	2.80 (2.34, 3.66)	2.40 (2.10, 3.19)	Z=-1.56	0.120
CEA(ng/mL), M (Q_1_, Q_3_)	1.44 (0.81, 1.84)	1.29 (0.79, 1.80)	1.54 (0.81, 1.95)	Z=-0.82	0.411
SCC(ng/mL), M (Q_1_, Q_3_)	1.17 (0.71, 1.67)	0.95 (0.64, 1.62)	1.22 (0.75, 1.85)	Z=-1.38	0.167
CA15-3(U/mL), M (Q_1_, Q_3_)	13.20 (7.40, 20.70)	12.95 (7.38, 21.05)	13.35 (7.47, 20.55)	Z=-0.03	0.974
HE4(pmol/L), M (Q_1_, Q_3_)	54.15 (51.10, 58.42)	55.10 (51.18, 58.47)	53.90 (51.10, 58.35)	Z=-0.41	0.681
CA125(U/mL), M (Q_1_, Q_3_)	13.20 (8.86, 17.70)	12.00 (8.79, 16.22)	13.50 (9.37, 18.25)	Z=-1.02	0.308
Maximum diameter(mm), Mean ± SD	21.19 ± 14.43	21.40 ± 15.73	21.10 ± 13.92	t=0.11	0.912
Tumor depth(mm), M (Q_1_, Q_3_)	6.20 (4.00, 8.30)	6.10 (3.70, 8.65)	6.30 (4.40, 8.25)	Z=-0.55	0.584
Peak systolic velocity, M (Q_1_, Q_3_)	11.00 (8.34, 16.98)	10.50 (7.90, 15.89)	11.50 (8.51, 17.07)	Z=-0.69	0.492
Resistance index, M (Q_1_, Q_3_)	0.68 (0.59, 0.78)	0.69 (0.54, 0.78)	0.68 (0.61, 0.78)	Z=-0.29	0.774
E_mean_, M (Q_1_, Q_3_)	287.26 (218.14, 350.67)	277.83 (206.79, 346.48)	291.18 (225.92, 357.52)	Z=-0.74	0.462
E_max_, M (Q_1_, Q_3_)	753.82 (530.13, 1070.97)	757.41 (502.45, 1257.52)	753.82 (566.53, 997.69)	Z=-0.19	0.849
E_min_, M (Q_1_, Q_3_)	74.20 (32.65, 116.40)	74.08 (35.67, 114.98)	74.20 (32.65, 119.60)	Z=-0.38	0.705
E_sd_, M (Q_1_, Q_3_)	123.08 (83.77, 167.19)	123.35 (85.07, 186.22)	123.08 (83.77, 165.84)	Z=-0.57	0.571
Ratio of elastic moduli, M (Q_1_, Q_3_)	4.06 (2.42, 6.77)	4.41 (2.47, 7.31)	3.92 (2.42, 6.57)	Z=-0.35	0.727
Elasticity scoring, M (Q_1_, Q_3_)	4.00 (3.00, 4.00)	4.00 (3.00, 4.00)	4.00 (3.00, 4.00)	Z=-1.30	0.192
Calcification, n(%)	-	-	-	-	1.000
No	66 (50.00)	20 (50.00)	46 (50.00)	-	-
Macrocalcification	5 (3.79)	1 (2.50)	4 (4.35)	-	-
Microcalcification	61 (46.21)	19 (47.50)	42 (45.65)	-	-
Heterogeneous echogenicity, n(%)	-	-	-	χ^2^=0.00	0.981
0	79 (59.85)	24 (60.00)	55 (59.78)	-	-
1	53 (40.15)	16 (40.00)	37 (40.22)	-	-
Blurry boundaries, n(%)	-	-	-	χ^2^=0.41	0.520
0	65 (49.24)	18 (45.00)	47 (51.09)	-	-
1	67 (50.76)	22 (55.00)	45 (48.91)	-	-
Irregular shape, n(%)	-	-	-	χ^2^=0.76	0.385
0	75 (56.82)	25 (62.50)	50 (54.35)	-	-
1	57 (43.18)	15 (37.50)	42 (45.65)	-	-
Liquefaction necrosis, n(%)	-	-	-	χ^2^=0.00	1.000
0	127 (96.95)	39 (97.50)	88 (96.70)	-	-
1	4 (3.05)	1 (2.50)	3 (3.30)	-	-
Penetrating vessels, n(%)	-	-	-	χ^2^=0.26	0.609
0	77 (58.33)	22 (55.00)	55 (59.78)	-	-
1	55 (41.67)	18 (45.00)	37 (40.22)	-	-
SRS, n(%)	-	-	-	χ^2^=0.52	0.470
0	82 (62.12)	23 (57.50)	59 (64.13)	-	-
1	50 (37.88)	17 (42.50)	33 (35.87)	-	-

**Note:** AFP: Alpha-Fetoprotein; CA125: Cancer Antigen 125; CA15-3: Cancer Antigen 15-3; CA199: Carbohydrate Antigen 199; CEA: Carcinoembryonic Antigen; HE4: Human Epididymis Protein 4; SCC: Squamous Cell Carcinoma Antigen; SRS: stiff rim sign.

**Table 2:** Comparison of clinical baseline data and B-mode ultrasound imaging characteristics of benign and malignant breast masses in the training set.

**Variables**	**Total (n = 92)**	**Benign (n = 42)**	**Malignant (n = 50)**	**Statistic**	***P***
Age, Mean ± SD	50.33 ± 11.76	51.07 ± 13.13	49.70 ± 10.57	t=0.56	0.580
BMI, Mean ± SD	22.90 ± 2.32	22.90 ± 2.15	22.90 ± 2.48	t=0.02	0.986
CA199(U/mL), M (Q_1_, Q_3_)	7.90 (5.30, 12.05)	7.02 (4.70, 11.30)	8.10 (6.38, 12.73)	Z=-1.41	0.159
AFP(ng/mL), M (Q_1_, Q_3_)	2.40 (2.10, 3.19)	2.45 (2.10, 3.20)	2.40 (2.12, 3.14)	Z=-0.09	0.928
CEA(ng/mL), M (Q_1_, Q_3_)	1.54 (0.81, 1.95)	1.12 (0.77, 1.83)	1.60 (1.02, 2.32)	Z=-1.81	0.070
SCC(ng/mL), M (Q_1_, Q_3_)	1.22 (0.75, 1.85)	1.22 (0.72, 1.64)	1.49 (0.75, 1.86)	Z=-0.49	0.624
CA15-3(U/mL), M (Q_1_, Q_3_)	13.35 (7.47, 20.55)	9.15 (6.40, 13.00)	16.80 (13.12, 23.20)	Z=-4.54	<.001
HE4(pmol/L), M (Q_1_, Q_3_)	53.90 (51.10, 58.35)	53.40 (51.35, 59.10)	54.45 (51.10, 58.10)	Z=-0.21	0.835
CA125(U/mL), M (Q_1_, Q_3_)	13.50 (9.37, 18.25)	11.85 (7.48, 15.90)	14.30 (11.00, 19.10)	Z=-2.09	0.036
Maximum diameter(mm), Mean ± SD	21.10 ± 13.92	21.05 ± 17.86	21.14 ± 9.63	t=-0.03	0.975
Tumor depth(mm), M (Q_1_, Q_3_)	6.30 (4.40, 8.25)	5.65 (4.75, 8.00)	6.70 (4.00, 9.00)	Z=-0.76	0.444
Peak systolic velocity, M (Q_1_, Q_3_)	11.50 (8.51, 17.07)	11.00 (7.90, 15.20)	12.41 (8.75, 17.15)	Z=-1.00	0.317
Resistance index, M (Q_1_, Q_3_)	0.68 (0.61, 0.78)	0.62 (0.56, 0.73)	0.71 (0.64, 0.78)	Z=-1.53	0.126
E_mean_, M (Q_1_, Q_3_)	291.18 (225.92, 357.52)	272.35 (202.74, 325.03)	311.80 (250.34, 378.11)	Z=-1.78	0.076
E_max_, M (Q_1_, Q_3_)	753.82 (566.53, 997.69)	611.70 (465.07, 871.65)	878.54 (663.26, 1067.32)	Z=-2.78	**0.005**
E_min_, M (Q_1_, Q_3_)	74.20 (32.65, 119.60)	74.20 (34.12, 104.74)	76.00 (26.94, 119.95)	Z=-0.09	0.928
E_sd_, M (Q_1_, Q_3_)	123.08 (83.77, 165.84)	106.91 (67.56, 144.70)	138.51 (100.23, 177.62)	Z=-2.64	**0.008**
Ratio of elastic moduli, M (Q_1_, Q_3_)	3.92 (2.42, 6.57)	2.50 (2.03, 3.29)	6.51 (5.00, 8.06)	Z=-6.80	**<.001**
Elasticity scoring, M (Q_1_, Q_3_)	4.00 (3.00, 4.00)	3.00 (3.00, 3.00)	4.00 (4.00, 4.00)	Z=-8.04	**<.001**
Calcification, n(%)	-	-	-	-	0.339
no	46 (50.00)	24 (57.14)	22 (44.00)	-	-
Macrocalcification	4 (4.35)	2 (4.76)	2 (4.00)	-	-
Microcalcification	42 (45.65)	16 (38.10)	26 (52.00)	-	-
Heterogeneous echogenicity, n(%)	-	-	-	χ^2^=0.65	0.419
no	55 (59.78)	27 (64.29)	28 (56.00)	-	-
yes	37 (40.22)	15 (35.71)	22 (44.00)	-	-
Blurry boundaries, n(%)	-	-	-	χ^2^=23.36	**<.001**
no	47 (51.09)	33 (78.57)	14 (28.00)	-	-
yes	45 (48.91)	9 (21.43)	36 (72.00)	-	-
Irregular shape, n(%)	-	-	-	χ^2^=4.73	**0.030**
no	50 (54.35)	28 (66.67)	22 (44.00)	-	-
yes	42 (45.65)	14 (33.33)	28 (56.00)	-	-
Liquefaction necrosis, n(%)	-	-	-	χ^2^=0.00	1.000
no	88 (96.70)	40 (97.56)	48 (96.00)	-	-
yes	3 (3.30)	1 (2.44)	2 (4.00)	-	-
Penetrating vessels, n(%)	-	-	-	χ^2^=30.28	**<.001**
no	55 (59.78)	38 (90.48)	17 (34.00)	-	-
yes	37 (40.22)	4 (9.52)	33 (66.00)	-	-
SRS, n(%)	-	-	-	χ^2^=12.39	**<.001**
no	59 (64.13)	35 (83.33)	24 (48.00)	-	-
yes	33 (35.87)	7 (16.67)	26 (52.00)	-	-

**Note:** AFP: Alpha-Fetoprotein; CA125: Cancer Antigen 125; CA15-3: Cancer Antigen 15-3; CA199: Carbohydrate Antigen 199; CEA: Carcinoembryonic Antigen; HE4: Human Epididymis Protein 4; SCC: Squamous Cell Carcinoma Antigen; SRS: stiff rim sign.

**Table 3:** Univariate logistic regression analysis of risk factors associated with malignant breast masses in the training set.

**Variables**	**β**	**S.E**	**Z**	***P***	**OR (95%CI)**
Blurry boundaries	-	-	-	-	-
no	-	-	-	-	1.00 (Reference)
yes	2.24	0.49	4.57	<.001	9.43 (3.61 ~ 24.66)
Irregular shape	-	-	-	-	-
no	-	-	-	-	1.00 (Reference)
yes	0.93	0.43	2.15	0.031	2.55 (1.09 ~ 5.96)
Penetrating vessels	-	-	-	-	-
no	-	-	-	-	1.00 (Reference)
yes	2.91	0.60	4.82	<.001	18.44 (5.64 ~ 60.30)
SRS	-	-	-	-	-
no	-	-	-	-	1.00 (Reference)
yes	1.69	0.50	3.37	<.001	5.42 (2.03 ~ 14.48)
E_max_	0.01	0.00	2.48	0.013	1.01 (1.01 ~ 1.01)
E_sd_	0.01	0.00	2.54	0.011	1.01 (1.01 ~ 1.02)
Ratio of elastic moduli	1.20	0.25	4.78	<.001	3.32 (2.03 ~ 5.42)
CA15-3(U/mL)	0.12	0.04	3.18	0.001	1.12 (1.05 ~ 1.21)
CA125(U/mL)	0.07	0.04	2.07	0.038	1.08 (1.01 ~ 1.15)
Elasticity scoring	4.99	0.79	6.28	<.001	146.32 (30.85 ~ 694.02)

**Note:** CA125: Cancer Antigen 125; CA15-3: Cancer Antigen 15-3; SRS: stiff rim sign.

**Table 4:** Multivariate logistic regression analysis of independent risk factors for malignant breast masses in the training set.

**Variables**	**β**	**S.E**	**Z**	**P**	**OR (95%CI)**
Intercept	-42.18	21.51	-1.96	0.050	0.00 (0.00 ~ 0.98)
Blurry boundaries	-	-	-	-	-
No	-	-	-	-	1.00 (Reference)
Yes	0.09	2.15	0.04	0.967	1.09 (0.02 ~ 74.03)
Irregular shape	-	-	-	-	-
No	-	-	-	-	1.00 (Reference)
Yes	1.18	2.75	0.43	0.667	3.26 (0.01 ~ 713.45)
Penetrating vessels	-	-	-	-	-
No	-	-	-	-	1.00 (Reference)
Yes	3.92	3.33	1.18	0.239	50.47 (0.07 ~ 34730.75)
SRS	-	-	-	-	-
No	-	-	-	-	1.00 (Reference)
Yes	-2.37	3.85	-0.61	0.539	0.09 (0.00 ~ 176.91)
E_max_	0.01	0.01	0.92	0.360	1.01 (0.99 ~ 1.03)
E_sd_	-0.02	0.05	-0.31	0.760	0.99 (0.89 ~ 1.09)
Ratio of elastic moduli	2.06	1.29	1.59	0.112	7.81 (0.62 ~ 98.26)
CA15-3(U/mL)	0.15	0.14	1.07	0.283	1.17 (0.88 ~ 1.54)
CA125(U/mL)	0.01	0.12	0.12	0.906	1.01 (0.80 ~ 1.28)
Elasticity scoring	7.50	3.67	2.04	0.041	1803.27 (1.36 ~ 2394498.67)

**Note:** CA125: Cancer Antigen 125; CA15-3: Cancer Antigen 15-3; SRS: stiff rim sign.

**Table 5:** ROC curve analysis for predicting diagnostic efficacy of the nomogram model.

Data	AUC (95%CI)	Accuracy (95%CI)	Sensitivity (95%CI)	Specificity (95%CI)	PPV vessels (95%CI)	NPV vessels (95%CI)	Cut Off
Train	1.00 (0.99-1.00)	0.98 (0.92-1.00)	1.00 (1.00 - 1.00)	0.96 (0.90 - 1.00)	0.95 (0.89 - 1.00)	1.00 (1.00 - 1.00)	0.711
Test	0.96 (0.91-1.00)	0.88 (0.73-0.96)	0.86 (0.67 - 1.00)	0.88 (0.76 - 1.00)	0.80 (0.60 - 1.00)	0.92 (0.81 - 1.00)	0.711

**Note:** AUC: Area Under the Curve; NPV: Negative Predictive Value; PPV: Positive Predictive Value.

## Data Availability

The data of current study are available from corresponding author, [B.L], on a reasonable request.

## LIST OF ABBREVIATIONS

AUC: Area Under the Curve
BMI: Body mass index
CEA: Carcinoembryonic antigen
DCA: Decision curve analysis
E_max_: Maximum Young’s modulus hardness value
E_mean_: Mean
E_min_: Minimum Young’s modulus hardness value
E_sd_: Standard deviation of Young’s modulus hardness value
MRI: Magnetic resonance imaging
ROC: Receiver Operating Characteristic
ROI: Region of interest
S_max_: Maximum Young’s modulus hardness value of the 2 mm shell
S_mean_: Mean Young’s modulus hardness value of the 2 mm shell
S_min_: Minimum Young’s modulus hardness value of the 2 mm shell
SRS: Stiff rim sign
S_sd_: Standard deviation of Young’s modulus hardness value of the 2 mm shell
XAI: Explainable Artificial Intelligence

## STANDARDS OF REPORTING

STROBE guidelines were followed.
